# Erratum: Chikako, S.; Yoshihisa, W.; Youichi, T.; Toshitaka, N. Influence of Housing Systems on Physical, Emotional, and Cognitive Functions with Aging in DBA/2CrSlc Mice *Animals* 2020, *10*, 746

**DOI:** 10.3390/ani10050813

**Published:** 2020-05-07

**Authors:** Chikako Shimizu, Yoshihisa Wakita, Youichi Tsuchiya, Toshitaka Nabeshima

**Affiliations:** 1 Frontier Laboratories for Value Creation, SAPPORO HOLDINGS LTD., 10 Okatome, Yaizu, Shizuoka 425-0013, Japan; Yoshihisa.Wakita@sapporoholdings.co.jp (Y.W.); Yoichi.Tsuchiya@sapporoholdings.co.jp (Y.T.); 2 Advanced Diagnostic System Research Laboratory, Fujita Health University, 1-98 Dengakugakubo, Kutsukake-cho, Toyoake, Aichi 470-1192, Japan; tnabeshi@ccalumni.meijo-u.ac.jp; 3 NPO Japanese Drug Organization of Appropriate Use and Research, 3-1509 Omoteyama, Tenpaku-ku, Nagoya, Aichi 468-0069, Japan

The authors wish to make the following corrections to their paper [1]:

In Table 1, Table 2 and Table 3, the values for the Repeated Measures Two-Way ANOVA and Two-Way ANOVA columns had typing errors and have now been corrected (see the corrected version of Table 1, Table 2 and Table 3 bellow):

## Figures and Tables

**Table 1:** Results of statistical analysis in Test 1–Test 3.

		Repeated Measures Two-Way ANOVA	Two-Way ANOVA
		Alive Mice at 96 Weeks of Age	All Mice
Balancing Ability on an Acryl Rod (Test 1)	age	*F* (11,253) = 20.0, *p* < 0.001	*F* (11,365) = 16.6, *p* < 0.001
	group	*F* (1,23) = 1.63, *p* > 0.05	*F* (1,365) = 14.8, *p* < 0.001
	age × group	*F* (11,253) =1.59, *p* > 0.05	*F* (11,365) = 1.04, *p* > 0.05
Horizontal Bar Test (Test 2)	age	*F* (11,253) = 16.0, *p* < 0.001	*F* (11,365) = 17.1, *p* < 0.001
	group	*F* (1,23) = 4.10, *p* = 0.054	*F* (1,365) = 30.5, *p* < 0.001
	age × group	*F* (11,253) = 1.00, *p* > 0.05	*F* (11,365) = 0.99, *p* > 0.05
Wire Hanging Test (Test 3)	age	*F* (11,253) = 11.0, *p* < 0.001	*F* (11,365) = 10.5, *p* < 0.001
	group	*F* (1,23) = 26.7, *p* < 0.001	*F* (1,365) = 179.0, *p* < 0.001
	age × group	*F* (11,253) = 1.00, *p* < 0.05	*F* (11,365) = 2.16, *p* < 0.05

**Table 2:** Results of statistical analysis in Test 4.

Foot Print Test (Test 4)		Repeated Measures Two-Way ANOVA	Two-Way ANOVA
		Alive Mice at 93 Weeks of Age	All Mice
Stride of forefoot	age	*F* (5,120) = 16.4, *p* < 0.001	*F* (5,182) = 18.0, *p* < 0.001
	group	*F* (1,24) = 16.4, *p* < 0.05	*F* (1,182) = 4.45, *p* < 0.05
	age × group	*F* (5,120) = 1.73, *p* > 0.05	*F* (5,182) = 1.85, *p* > 0.05
Sway of forefoot	age	*F* (5,120) = 10.8, *p* < 0.001	*F* (5,182) = 12.2, *p* < 0.001
	group	*F* (1,24) = 4.15, *p* = 0.051	*F* (1,182) = 3.86, *p* = 0.051
	age × group	*F* (5,120) = 0.70, *p* > 0.05	*F* (5,182) = 1.56, *p* > 0.05
Stance of forefoot	age	*F* (5,120) = 2.48, *p* < 0.05	*F* (5,182) = 3.29, *p* < 0.01
	group	*F* (1,24) = 0.245, *p* > 0.05	*F* (1,182) = 0.0007, *p* > 0.05
	age × group	*F* (5,120) = 1.30, *p* > 0.05	*F* (5,182) = 1.16, *p* > 0.05
Stride of hindfoot	age	*F* (5,120) = 8.51, *p* < 0.001	*F* (5,182) = 9.06, *p* < 0.001
	group	*F* (1,24) = 14.4, *p* < 0.001	*F* (1,182) = 8.26, *p* < 0.01
	age × group	*F* (5,120) = 1.19, *p* > 0.05	*F* (5,182) = 1.08, *p* > 0.05
Sway of hindfoot	age	*F* (5,120) = 2.70, *p* < 0.05	*F* (5,182) = 4.65, *p* < 0.001
	group	*F* (1,24) = 13.4, *p* < 0.01	*F* (1,182) = 10.9, *p* < 0.01
	age × group	*F* (5,120) = 2.01, *p* > 0.05	*F* (5,182) = 1.31, *p* > 0.05
Stance of hindfoot	age	*F* (5,120) = 4.56, *p* < 0.01	*F* (5,182) = 3.39, *p* < 0.01
	group	*F* (1,24) = 0.85, *p* > 0.05	*F* (1,182) = 1.59, *p* > 0.05
	age × group	*F* (5,120) = 1.37, *p* > 0.05	*F* (5,182) = 1.50, *p* > 0.05

**Table 3:** Results of statistical analysis in Test 5.

Locomotor Activity (Test 5)		Repeated Measures Two-Way ANOVA	Two-Way ANOVA
		Alive Mice at 96 Weeks of Age	All Mice
Locomotor activity	age	*F* (8,168) = 8.75, *p* < 0.001	*F* (8,268) = 6.76, *p* < 0.001
	group	*F* (1,21) = 0.032, *p* > 0.05	*F* (1,268) = 0.083, *p* > 0.05
	age × group	*F* (8,168) = 0.95, *p* > 0.05	*F* (8,268) = 0.554, *p* > 0.05
Center time	age	*F* (8,168) = 29.0, *p* < 0.001	*F* (8,268) = 26.0, *p* < 0.001
	group	*F* (1,21) = 1.20, *p* > 0.05	*F* (1,268) = 0.544, *p* > 0.05
	age × group	*F* (8,168) = 4.20, *p* < 0.01	*F* (8,268) = 2.83, *p* < 0.001
