# Supplementary material for: Influence of Housing Systems on Physical, Emotional, and Cognitive Functions with Aging in DBA/2CrSlc Mice
Source: Animals (Basel). 2020 Apr 24;10(4):746. doi: 10.3390/ani10040746 (PMC7222825; doi:10.3390/ani10040746)
Supplement: Supplementary file 1 [file animals-10-00746-s001.zip › Figure S1 Champing behaviors to slice papers of bedding in cages and dishcloth gourd for the enrichment in the Chamber and IVC groups.docx]

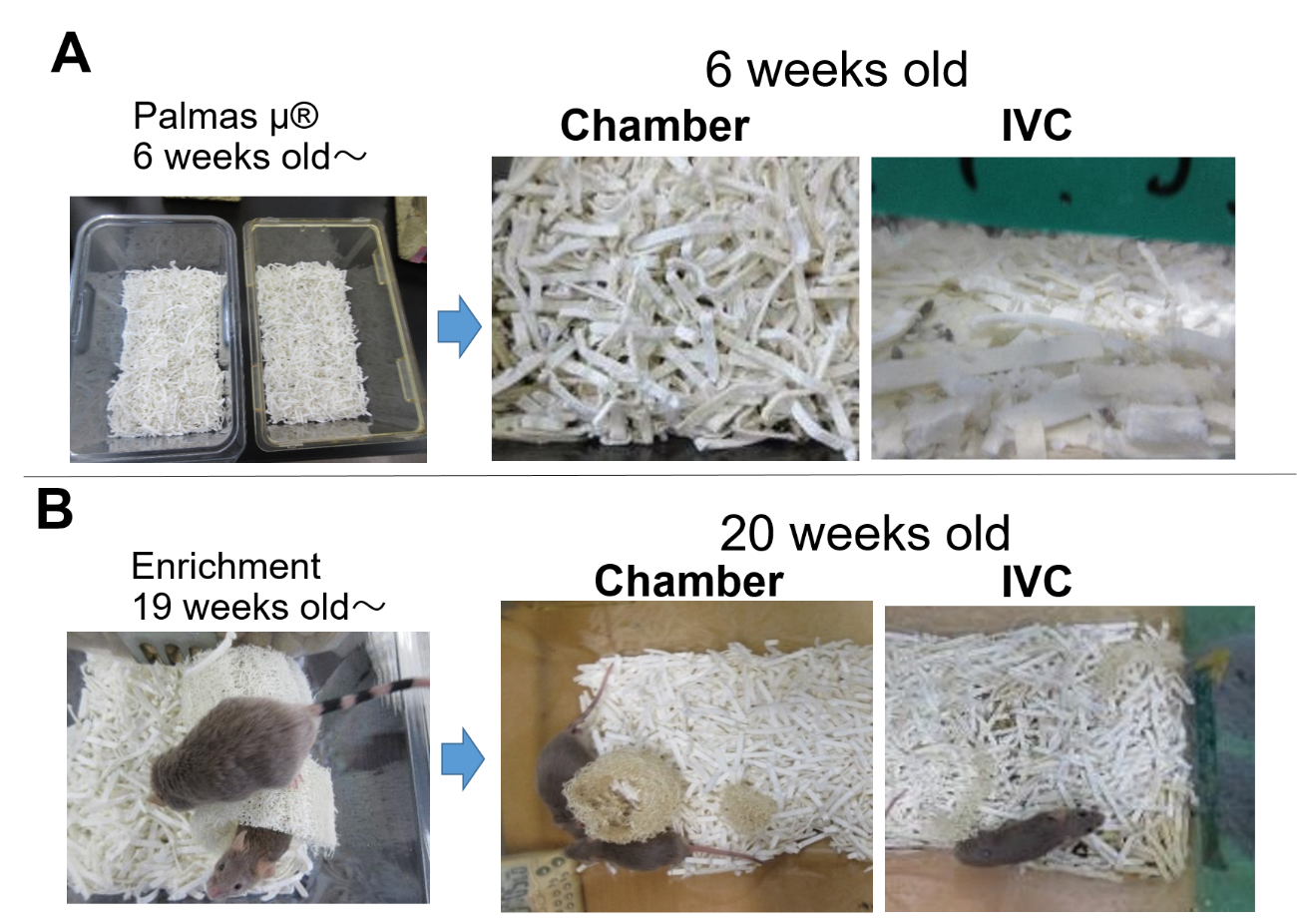


Figure S1: Champing behaviors to slice papers of bedding in cages and dishcloth gourd for the enrichment in cage in the Chamber and IVC groups.

(A) conditions of slice papers of bedding in cages in both groups at the age of 6 weeks;

(B) conditions of dishcloth gourd in cages in both groups at the age of 20 weeks.
