# Supplementary material for: Influence of Housing Systems on Physical, Emotional, and Cognitive Functions with Aging in DBA/2CrSlc Mice
Source: Animals (Basel). 2020 Apr 24;10(4):746. doi: 10.3390/ani10040746 (PMC7222825; doi:10.3390/ani10040746)
Supplement: Supplementary file 1 [file animals-10-00746-s001.zip › Figure S2 Changes in the bulge of the rump in the Chamber and IVC groups at the age of 94 weeks.docx]

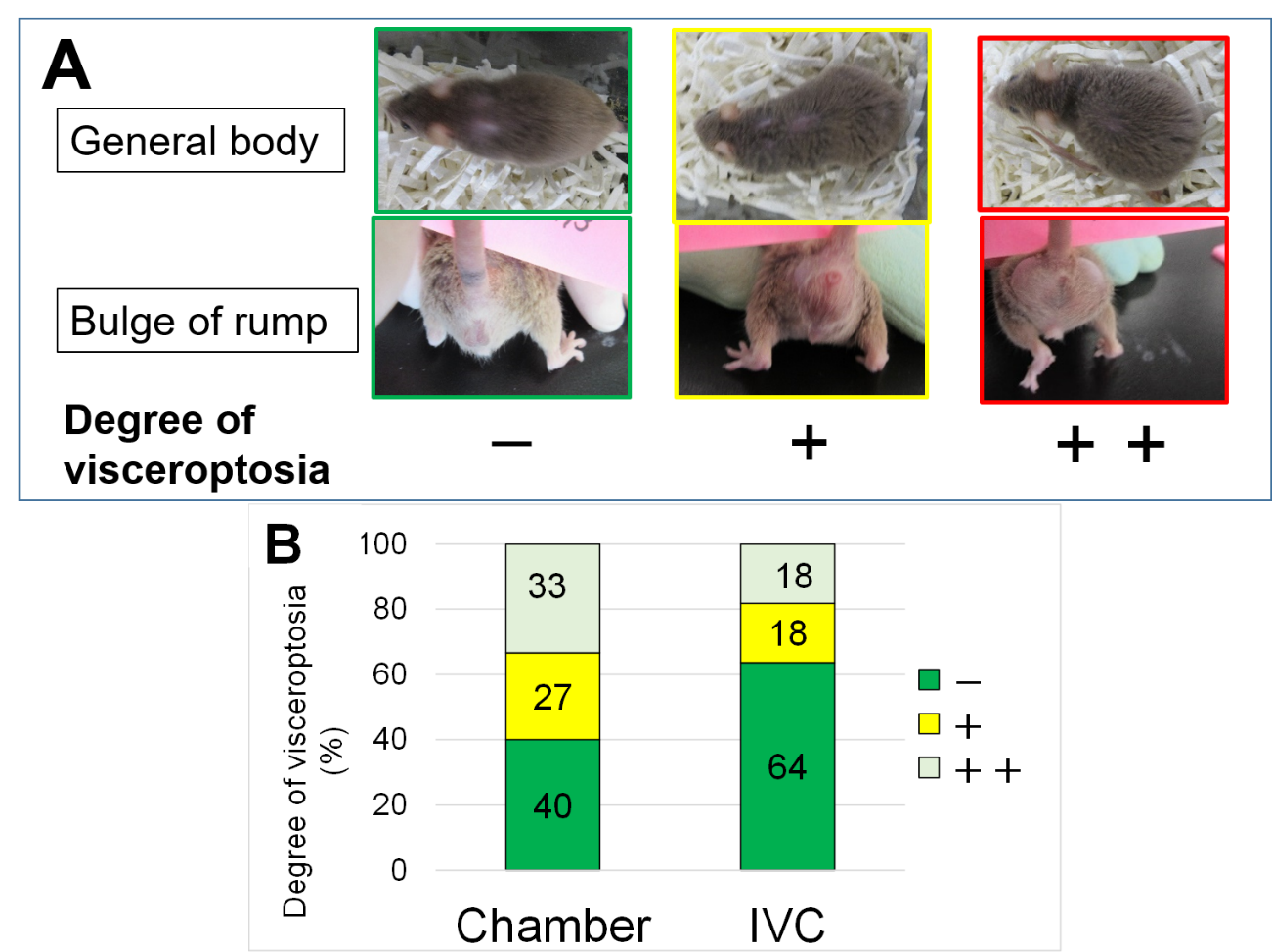


Figure S2: Changes in the bulge of rump in the Chamber and IVC groups at the age of 94 weeks.

(A) three degrees (-, +, ++) of visceroptosia (upper: general body, downer: bulge of rump):

(B) ratio of three degrees of visceroptosia in the Chamber and IVC groups.
