# Supplementary material for: Influence of Housing Systems on Physical, Emotional, and Cognitive Functions with Aging in DBA/2CrSlc Mice
Source: Animals (Basel). 2020 Apr 24;10(4):746. doi: 10.3390/ani10040746 (PMC7222825; doi:10.3390/ani10040746)
Supplement: Supplementary file 1 [file animals-10-00746-s001.zip › Table S1 Protocols of experiments for physical, emotional, cognitive functions in the Chamber and IVC groups.docx]

**Table S1** Protocols of experiments for physical, emotional, cognitive functions in the Chamber and IVC groups
